# The *Framing* of machine learning risk prediction models illustrated by evaluation of sepsis in general wards

**DOI:** 10.1038/s41746-021-00529-x

**Published:** 2021-11-15

**Authors:** Simon Meyer Lauritsen, Bo Thiesson, Marianne Johansson Jørgensen, Anders Hammerich Riis, Ulrick Skipper Espelund, Jesper Bo Weile, Jeppe Lange

**Affiliations:** 1 Enversion A/S, Fiskerivej 12, 1st floor, 8000 Aarhus C, Denmark; 2 Department of Clinical Medicine, Aarhus University, Aarhus N, Denmark; 3 Department of Engineering, Aarhus University, Aarhus C, Denmark; 4 Department of Research, Horsens Regional Hospital, Horsens, Denmark; 5 Department of Anesthesiology, Horsens Regional Hospital, Horsens, Denmark; 6 Emergency Department, Horsens Regional Hospital, Horsens, Denmark; 7 Research Center for Emergency Medicine, Aarhus University Hospital, Aarhus, Denmark

**Keywords:** Machine learning, Epidemiology, Biomedical engineering, Computer science

## Abstract

Problem framing is critical to developing risk prediction models because all subsequent development work and evaluation takes place within the context of how a problem has been framed and explicit documentation of framing choices makes it easier to compare evaluation metrics between published studies. In this work, we introduce the basic concepts of framing, including prediction windows, observation windows, window shifts and event-triggers for a prediction that strongly affects the risk of clinician fatigue caused by false positives. Building on this, we apply four different framing structures to the same generic dataset, using a sepsis risk prediction model as an example, and evaluate how framing affects model performance and learning. Our results show that an apparently good model with strong evaluation results in both discrimination and calibration is not necessarily clinically usable. Therefore, it is important to assess the results of objective evaluations within the context of more subjective evaluations of how a model is framed.

## Introduction

The first stage in developing any machine learning risk prediction (MLRP) model in healthcare is formulating what needs to be predicted and how to define it (a clinical event such as sepsis). This is, by itself, not an easy task, given that many clinical events for which MLRP are suitable, are based on composite and or collapsed data events. More so, many clinical events, when experienced in real-life healthcare settings are based on expert consensus-opinions (such as Sequential Organ Failure Assessment (SOFA) guidelines for sepsis^1,2^). These data events also originate from complete, or not-so-complete, electronic healthcare registers with divergent availability (respiration frequency in intensive care units (ICU) vs. general wards) and validity (blood samples vs. administrative codes) of data.

However, when formulating what clinical event needs to be predicted, it is also crucial to define the temporal aspects of this prediction and, in particular, when and how often one needs this prediction to occur for the healthcare staff to act on it. This aspect of a precise formulation of a healthcare MLRP model has so far not been readily recognized in the published literature. Here, an in-depth clinical understanding of the clinical event, the complexity of the definition of the clinical event, and the temporal aspect of the model in relation to the clinical event being predicted, is required, as modeling choices made at this stage will shape the rest of the development process.

In this article, we refer to the collection of the above concepts (defining the clinical event and the temporal aspects) at this stage of model development as “framing the MLRP problem” with focus on the temporal aspects. Framing the MLRP problem is thus a task which needs a high degree of cooperation between the data scientist constructing the model and the end-users, the clinician, for which the MLRP is intended, at the earliest possible time of model development. A common language is required to optimize this process.

We have chosen to illustrate the temporal aspect of the framing with an MLRP model that aims to predict the onset of a clinical event—sepsis. The temporal aspect of predicting the clinical event in time can be formulated in various ways, here represented as questions for the MLRP model to solve: Will this patient experience the clinical event (1) within the next 12 h, (2) in exactly 12 h, (3) within 8–12 h, (4) during this admission, (5) during an anticipated operation, or (6) within 30 days from discharge? Additionally, this question can be stated at different frequencies, such as each hour, at hourly intervals or once per day, or at particular points in time during an admission.

In this article, we demonstrate the consequences of different temporal aspect framings of the MLRP problem. The goal is illustrate why the framing of the MLRP model is critically important, when presenting developed models in healthcare literature with suggestions to include aspects of this in the TRIPOD statements. We furthermore hope to create a common language for any scientist—data or medical, involved in the framing of an MLRP model. Finally, we present a quick guide to developers, as an inspiration to future MLRP model construction and reporting.

**Fig. 1:**
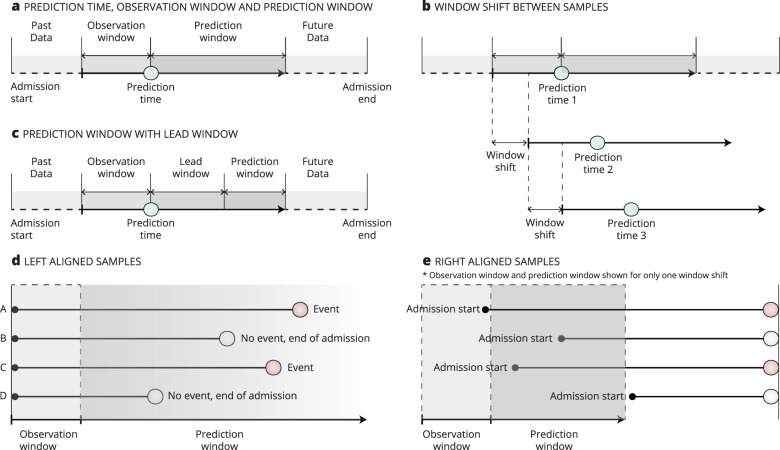
Basic concepts and terms of framing. **a** Illustrates prediction time, observation window, and prediction window. Window shift (**b**) is the distance between each prediction. **c** Shows a variation of (**a**) where the prediction window does not begin immediately after the prediction time but instead shifts to the lead window. **d** Shows an example of left-aligned samples, where the model is used for predicting fixed time points, such as admission or preoperatively. **e** Shows an example of right-aligned samples where all data are considered until some time point before the event of interest (for the positive cases) or the end of admission (for the negative cases).

Before proceeding, we introduce some of the constructions used to define the temporal aspect in the framing of the MLRP model. Figure 1 shows these constructions that form the temporal *framing structures* which can be applied for discussing the framing of the MLRP model.

- Prediction time. The time of prediction. This is when the model is used for predictions.
- Observation window. The period from which the independent variables, also called the explanatory variables, parameters, predictors, or features, are sampled. It is a retrospective window back in time as measured from the prediction time. The size of the observation window can have a fixed length for all samples or have varying lengths to reflect how much data are available for each sample.
- Prediction window. The period from which the dependent variable, also called the target, outcome, or event, is sampled.
- Lead time. The prediction window can start immediately after the prediction time (Fig. 1a) or be delayed (Fig. 1c). A prediction delay is called the *lead window* or gap window.
- Window shift. The shift is made between predictions, such as every hour. Window shift can have the same duration as a timestep, so the two are effectively the same in this case. This happens when, for example, an MLRP model makes a prediction every hour and where the observation window is simultaneously divided into timesteps of 1 h.
- Left alignmen**t**. When the samples in a dataset are left aligned, all samples are aligned by some specific event, such as admission or consultation, which also serves as the prediction time.
- Right alignment. When samples are right aligned, all data are considered until some duration before the event of interest (for the positive cases) or at the end of admission for the negative samples. Samples are aligned relative to the outcome of interest.

These constructs can be combined to form different temporal framing structures, such as survival analysis, a sliding window approach, and at-event framing. A survival analysis is a time-to-event analysis used to investigate the length of time until the occurrence of some well-defined event of interest, such as mortality. It is centered around a shared prediction time, called the time origin, which could be the time point of the diagnosis of cancer or onset of some disease^3^. A survival analysis imposes no specific requirements on the observation window, nor does it make use of a lead window. The prediction window starts immediately following the time origin but is subject to a variable length, due to censored data^3^ (Supplementary Fig. 1a).

The sliding window approach converts a sequential supervised learning problem into a standard supervised learning problem. The sliding window approach can be used to turn each window shift into independent samples that can be solved using any standard ML algorithm. Supplementary Figure 1b shows how multiple samples were generated with this approach. As with a survival analysis, the sliding window approach imposes no specific requirements on the observation window but can be implemented with a lead window, depending on the clinical problem to be solved.

Finally, in an at-event framing, the prediction is made at only one specific point in time, such as discharge. Being left aligned, the model is built to answer one question at just one specific time, such as the risk of 30-day readmission, so it does not include any window shifts (Supplementary Fig. 1c).

Building on this general introduction to the temporal aspect of framing, we turn our attention toward sepsis prediction. Sepsis prediction serves as an excellent illustrative case towards the necessity of framing the MLRP model, because it is a very popular area of research, and suffers from great variation in approaches to framing. As such, it was recently highlighted that structural heterogeneity of published sepsis prediction models limits pooling of overall performance^4,5^.

In the following, we have tried to place published models in different framing structures to make clear how different they are (Table 1). Most studies report right-aligned models, but there are great variations in problem framing even within this category. Barton et al. and Lauritsen et al.^6,7^ both used fixed time to onset structure where the prediction time were set to a specific duration before sepsis onset. In both studies, patients who had never developed sepsis were assigned an onset time randomly according to a continuous, uniform probability distribution. Scherpf et al. and Moor et al.^8,9^ both framed the sepsis prediction problem as a sequential problem solved with long short-term memory (LSTM)^10^ and temporal convolutional (TCN) networks^11,12^. Futoma et al. also framed the prediction problem as a sequential problem; given a new patient encounter, their goal was to continuously update the predicted probability that an encounter would result in sepsis at any time during admission^13^. As such, they did not predict whether sepsis would occur within a fixed prediction window but instead if the admission would result in sepsis at any time. Wyk et al. used a sliding window approach in which samples were generated by sliding the observation and prediction windows with small shifts in time^14^. Nemati used a similar sliding window approach^15^ and predicted for each shift in time if sepsis onset would occur within a fixed prediction window—which is in contrast to Futoma et al. In a later work by Futoma et al., the authors employed a more sophisticated and upgraded version of the fixed time to onset structure^16^. Khojandi et al. used both^17^ left- and right-aligned models. For their left-aligned model, they predicted the risk of sepsis at 12, 24, and 48 h after admission. For their right-aligned model, the authors predicted sepsis onset 12, 24, and 48 h before onset, with random sampled negative onset times. Khoshnevisan et al. used a fixed time to onset, but for the negative cases, instead of uniform sampling, the authors used the last registered timestamp in the admission as the onset time^18^.

**Table 1:** Framing structures in related studies.

Paper	Population	Target	Framing structure (1)	Alignment
Van Wyk, 2018^14^	Intensive care unit	Sepsis	Sliding window	Right
Scherpf, 2019^8^	Intensive care unit	Sepsis	Sequential problem	Right
Moor, 2019^9^	Intensive care unit	Sepsis	Sequential problem	Right
Futoma I, 2017^13^	Mixed ward	Sepsis	Sequential problem (modified)	Right
Futoma II, 2017^16^	Mixed ward	Sepsis	Fixed time to onset + matching	Right
Lauritsen I, 2020^19^	Mixed ward	Sepsis	Sequential problem	Right
Lauritsen II, 2020^6^	Mixed ward	Sepsis	Fixed time to onset	Right
Nemati, 2018^15^	Intensive care unit	Sepsis	Sliding window	Right
Delahanty, 2019^20^	Emergency department	Sepsis	On clinical demand (modified)	Right
Khojandi, 2018^17^	In hospital	Sepsis	Fixed time to onset, fixed time	Left & right
Khoshnevisan, 2018^18^	In hospital	Septic shock	Fixed time to onset (modified)	Left & right
Thiel, 2019^21^	In hospital	Septic shock	Fixed time to onset (modified)	Right
Van Wyk I, 2018^22^	Intensive care unit	Sepsis	Sliding window	Right
Wang, 2018^23^	Intensive care unit	Sepsis	Fixed time to onset	Right
Kam, 2017^24^	Intensive care unit	Sepsis	Sliding window	Right
Moss, 2016^25^	Intensive care unit	Severe sepsis	Sliding window	Right
Guillen, 2015^26^	Intensive care unit	Severe sepsis	Fixed time to onset	Right
Mao, 2017^27^	Mixed ward	Sepsis, severe sepsis, septic shock	Fixed time to onset	Right
Barton, 2019^7^	Mixed ward	Sepsis	Fixed time to onset	Right

(1) Details on framing structures are given in “framing of the prediction model” section and in the supplementary information.

In the current work, we generalize the framing structures identified in the literature to eight structures, from which we select four to implement in our analysis. We then apply five different ML algorithms and evaluate how discrimination and calibration vary between the four implemented framing structures. In a secondary analysis, we explore the differences in feature importance and feature interpretations between framing structures. Based on these analyses, we show that the MLRP model, which is superior in terms of discrimination and calibration, has little or no clinical value because of the way it is framed.

## Results

### Evaluations

In Table 2, the evaluation of the 4 framing structures and 5 ML algorithms is presented in summary form with mean values and 95% confidence intervals (CIs) over the 5 cross-validations.

**Table 2:** Evaluation results (mean values and 95% confidence intervals in brackets).

Model	AUPRC	AUROC	Brier *100 (y = 1)	Brier *100 (y = 0)	ACE (%)
Fixed time to onset					
Extra trees classifier	0.466 (0.435–0.496)	0.906 (0.897–0.915)	67.574 (65.277–69.870)	0.322 (0.286–0.358)	39.18 (35.76–42.60)
Random forest classifier	0.449 (0.407–0.491)	0.860 (0.852–0.869)	66.446 (64.476–68.417)	0.402 (0.374–0.430)	42.82 (38.12–47.52)
Light gradient boosting machine	0.317 (0.270–0.363)	0.815 (0.802–0.829)	74.290 (72.327–76.252)	0.415 (0.381–0.449)	45.35 (43.43–47.28)
XGBoost	0.292 (0.244–0.339)	0.777 (0.750–0.804)	78.062 (75.776–8.3470)	0.326 (0.293–0.358)	43.57 (42.24–44.90)
Logistic regression	0.239 (0.212–0.266)	0.752 (0.739–0.764)	77.662 (76.415–78.909)	0.466 (0.433–0.498)	46.63 (45.5–47.760)
Sliding windows					
Extra trees classifier	0.006 (0.005–0.007)	0.566 (0.539–0.593)	98.102 (97.811–98.394)	0.012 (0.011–0.013)	66.60 (63.21–69.98)
Random forest classifier	0.007 (0.007–0.008)	0.612 (0.604–0.621)	97.381 (97.170–97.592)	0.014 (0.013–0.015)	73.74 (73.06–74.43)
Light gradient boosting machine	0.004 (0.004–0.005)	0.624 (0.596–0.653)	98.785 (98.569–98.998)	0.015 (0.013–0.018)	75.22 (75.00–75.44)
XGBoost	0.007 (0.006–0.008)	0.756 (0.741–0.771)	98.852 (98.628–99.077)	0.003 (0.003–0.004)	75.14 (73.95–76.34)
Logistic regression	0.004 (0.004–0.004)	0.703 (0.688–0.717)	99.484 (99.462–99.506)	0.001 (0.001–0.001)	79.02 (73.57–84.47)
Sliding windows w. D.I.					
Extra trees classifier	0.009 (0.007–0.010)	0.593 (0.582–0.604)	97.580 (97.116–98.045)	0.016 (0.014–0.018)	64.56 (59.48–69.63)
Random forest classifier	0.011 (0.009–0.013)	0.638 (0.625–0.0651)	96.610 (96.006–97.213)	0.020 (0.018–0.022)	72.66 (71.22–74.10)
Light gradient boosting machine	0.007 (0.006–0.008)	0.665 (0.628–0.703)	98.278 (97.859–98.698)	0.023 (0.021–0.025)	74.89 (74.21–75.57)
XGBoost	0.011 (0.009–0.013)	0.747 (0.740–0.0755)	98.370 (98.180–98.560)	0.005 (0.004–0.005)	72.77 (70.57–74.96)
Logistic regression	0.006 (0.005–0.007)	0.684 (0.656–0.713)	99.285 (99.206–99.364)	0.001 (0.001–0.001)	82.13 (76.74–87.52)
On clinical demand					
Extra trees classifier	0.147 (0.139–0.155)	0.719 (0.704–0.733)	89.654 (89.217–9.090)	0.013 (0.012–0.014)	41.60 (38.40–44.80)
Random forest classifier	0.192 (0.154–0.231)	0.742 (0.717–.0766)	86.881 (85.482–88.281)	0.017 (0.016–0.017)	42.90 (39.20–46.60)
Light gradient boosting machine	0.056 (0.040–0.072)	0.774 (0.751–0.797)	91.376 (89.983–92.769)	0.030 (0.024–0.036)	62.89 (58.76–67.03)
XGBoost	0.114 (0.081–0.148)	0.779 (0.752–0.806)	91.799 (9.565–93.034)	0.009 (0.008–0.009)	46.79 (41.90–51.68)
Logistic regression	0.014 (0.011–0.016)	0.735 (0.720–0.749)	98.370 (98.167–98.572)	0.005 (0.005–0.006)	75.44 (74.25–76.63)
Best scoring machine learning models across framing structures and evaluation metrics					
Fixed time to onset	Extra trees	Extra trees	Random forest	Extra trees	Extra trees
Running windows	Random forest	XGBoost	Random forest	Logistic regression	Extra trees
Running windows w. D.I.	Random forest	XGBoost	Random forest	Logistic regression	Extra trees
On clinical demand	Random forest	XGBoost	Random forest	Logistic regression	Extra trees

AUPRC: Area under the precision recall curve; AUROC: Area under the receiver operating characteristics curve; Brier *100 (y = 1): The stratified Brier score for the positive class multiplied by 100; Brier *100 (y = 0): The stratified Brier score for the negative class multiplied by 100; ACE: Average calibration error; Sliding windows w. D.I.: Sliding window with dynamic inclusion.

The fixed time to onset framing achieved the best between-group discrimination results, with the highest AUPRC (0.466 (0.435–0.496)) and AUROC (0.906 (0.897–0.915)). It also achieved the best between-groups calibration with the lowest ACE of 39.18 (35.76–42.60). In the stratified Brier analysis, it achieved the best between-group score of 66.446 (64.476–68.417) for the positive class while having the worst between-group score for the negative class of 0.322 (0.286–0.358).

**Fig. 2:**
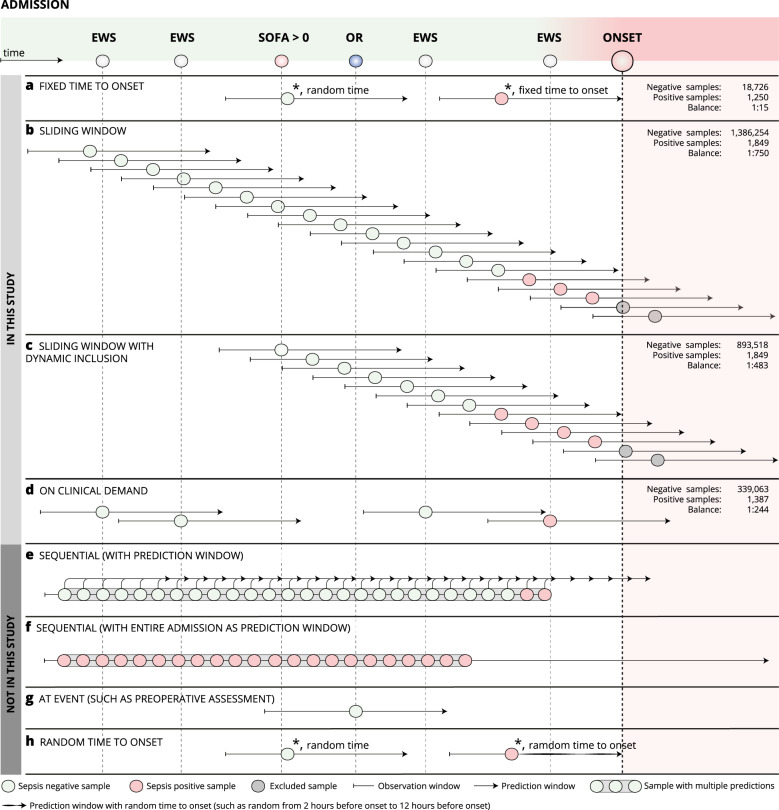
Framing of the machine learning algorithm. Eight different structures to framing the sepsis prediction model are shown. Fixed time to onset (**a**), sliding window (**b**), sliding window with dynamic inclusion (**c**), on clinical demand (**d**), sequential with prediction window (**e**), sequential with entire admission as prediction window (**f**), at event (**g**), and random time to onset (**h**). The top four structures (**a–d**) were explored, and the bottom four (**e–h**) were not. * indicates that the samples were collected from two different patients/admissions. EWS early warning score, SOFA sequential organ failure assessment scores, OR operating room.

As expected, the AUPRC varied widely as a result of the changing class balance across framing structures (Fig. 2). With a class balance of 1:750, the sliding window structure yielded the worst between-group AUPRC of 0.007 (0.007–0.008). This result was achieved by the best within-group extra trees classifier.

Similarly, the ACE and Brier scores varied greatly between framing structures, and the results followed the rank ordering dictated by class imbalance, such that both the ACE and Brier scores for the positive class decreased in the following order: fixed time to onset (class balance of 1:15), on clinical demand (class balance of 1:244), sliding window (class balance of 1:483) with dynamic inclusion, and sliding window (class balance of 1:750).

Brier scores for the negative class followed the opposite pattern, with the best within-class score of 0.322 (0.286–0.358) in the fixed time to onset and the best within-class score of 0.001 (0.001–0.001) with the sliding window approach.

The only evaluation metric not severely affected by class imbalance was the AUROC, which was expected because it plots true positive rate against false positive rate. As mentioned above, data from the fixed time to onset had superior performance of 0.906 (0.897–0.915), but the other three framing structures had an AUROC similar to each other with scores of 0.756 (0.741–0.771), 0.747 (0.740–0.755), and 0.779 (0.752–0.806) for the sliding window, sliding window with dynamic inclusion, and on clinical demand, respectively.

The lower part of Table 2 shows the best scoring ML models across framing structures and evaluation metrics. There is a tendency toward specific models dominating specific evaluations metrics. Random forest achieves the best AUPRC in three out of four framing structures. XGBoost has similar dominance regarding the AUROC. Random forest has the best Brier score for positive cases in all framing structures. A logistic regression has the best Brier score for negative cases in three out of four framing structures, and Extra Trees has the better ACE score for all framing structures.

**Fig. 3:**
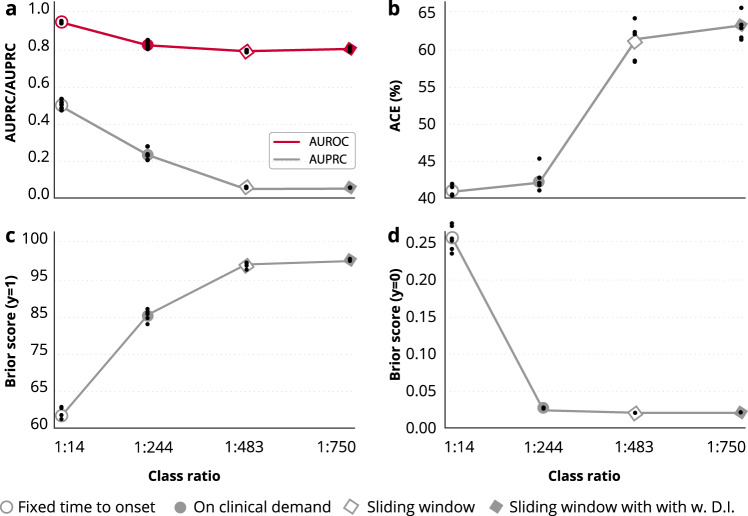
Relationship between the class ratio and evaluation results. The figure shows the mean values (circle or diamond) and individual cross-validation values (dots) for the best within-class MLRP model for each framing structures. **a** AUPRC (gray) and AUROC (red) discrimination results; (**b**) ACE; (**c**) The stratified Brier score for the positive class; (**d**) The stratified Brier score for negative class. AUPRC area under the precision recall curve, AUROC area under the receiver operating characteristics curve, ACE average calibration error.

Figure 3 shows the relationship between the class ratio and evaluation results. The figure shows the mean values for the best within-class MLRP model for each framing structure (as listed in Table 2). Individual results forming the basis of the mean values are plotted as small black dots. All five curves show monotonically decreasing or increasing behavior in relation to class ratio changes.

### Secondary analysis

**Fig. 4:**
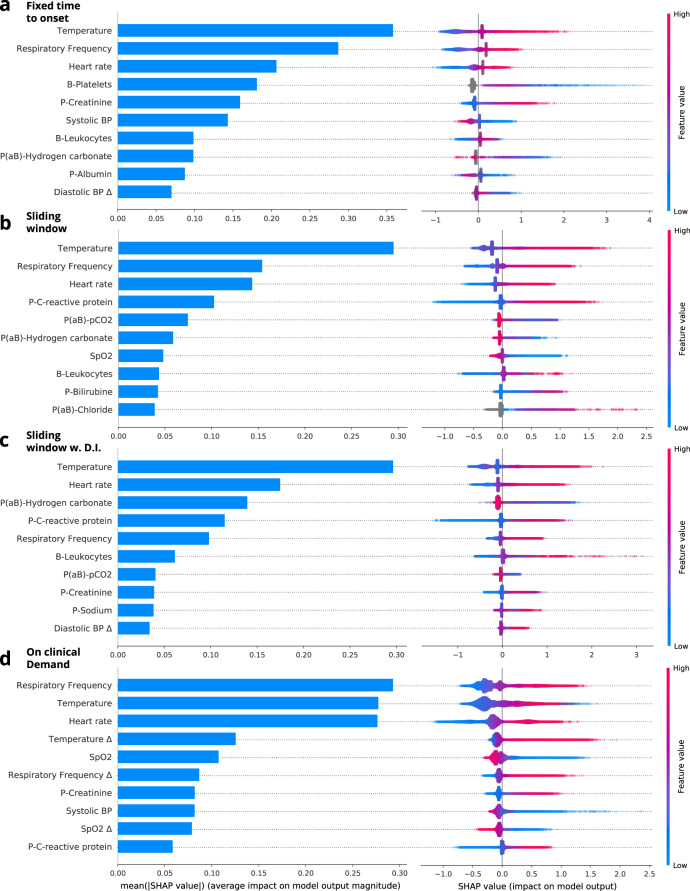
SHAP explanations. The blue. Fixed time to onset is shown in (**a**), sliding window in (**b**), sliding window with dynamic inclusion in (**c**), and on clinical demand in (**d**).

In Fig. 4, the 10 most important clinical parameters for each model of the four framing structures are shown. The parameters are sorted by decreasing means of the absolute SHAP values for all individuals in the dataset. The blue horizontal bars in the left column of Fig. 4 show the mean absolute SHAP values. The local explanation summary in the right-hand column of Fig. 4, represent the distributions of SHAP values across the explanation for individual samples in the dataset. These SHAP values are shown for each clinical parameter and color coded by the parameter value associated with the local explanation. Table 3, and Supplementary Table 1, shows the percentage of the missing values for the vital sign parameters for each of the four framing structures. Here, the percentage of missing values is significantly lower in the on clinical demand than in the other three framing structures.

**Table 3:** Missing values for vital sign parameters.

Parameter	Fixed time to onset (%)	Sliding window (%)	Sliding window w. D.I (%)	On clinical demand (%)
Heart rate	36.61	36.61	34.47	0.00
Respiratory frequency	38.85	38.85	36.81	0.00
SpO_2_	36.70	36.70	34.27	0.00
Systolic BP	36.45	36.45	34.29	0.00
Diastolic BP	36.51	36.51	34.36	0.07
Temperature	42.62	42.62	40.52	5.88
Heart rate Δ	80.86	80.86	78.30	26.81
Respiratory frequency Δ	81.42	81.42	78.96	26.81
SpO_2_ Δ	80.91	80.91	78.30	26.81
Systolic BP Δ	80.79	80.79	78.21	26.81
Diastolic BP Δ	80.80	80.80	78.22	26.85
Temperature Δ	82.42	82.42	79.99	30.95

**Fig. 5:**
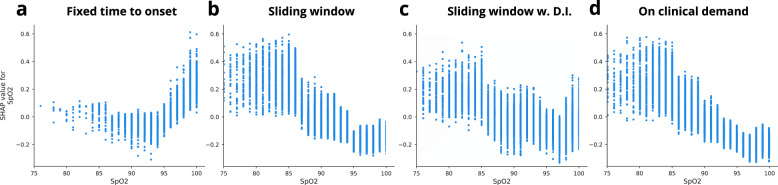
SHAP dependence plot SpO_2_. Fixed time to onset is shown in (**a**), sliding window in (**b**), sliding window with dynamic inclusion in (**c**), and on clinical demand in (**d**).

Figure 5 shows the dependence between the SpO_2_ values and the associated SpO_2_ SHAP values for the entire dataset. Here, the dependence curve is different for the fixed time to onset framing structure than for the other three framing structures, which follow a similar dependence curve. In the fixed time to onset framing structure, higher SpO_2_ values are associated with higher SHAP values, hence indicating a higher probability of sepsis.

## Discussion

We have shown how an MLRP model, when applied to a dataset obtained from general medical and surgical departments, can be temporal framed in different ways, and how the overall framing of the MLRP model has direct consequences for both the discriminative performance and calibration of the model as well as the associations learned by the model.

In our analysis, fixed time to onset achieved the best between-group discrimination results, showing the highest AUPRC and AUROC; it also achieved the best between-group calibration with the lowest ACE of 39.18 (35.76–42.60). At first glance, it may seem that this model is the best of the four models. However, it is important to assess the objective evaluation results in the context of a more subjective evaluation of the way in which the model is framed. The fixed time to onset structure does not represent a solid clinical question because the framing structure depends on future information. This inconsistency was highlighted by Wiens et al., who pointed out that evaluating a model at a specific point in time poses at least two issues: the need to define an onset time for the negative patients^19^. The fixed time to onset result does not provide results where the absolute numbers for sensitivity and specificity, at a given threshold, can be interpreted clinically. The fixed time to onset structure can be described as a naive case-control experiment where controls are samples randomly within some temporal constraints. The temporal bias of such methods are described in more detail in recent work by Yuan et al.^20^.

The results from Table 2 show that no one MLRP model was superior on all points. There was a tendency toward specific models dominating specific evaluations metrics. For example, extra trees had the best ACE score for all four framing structures. Similarly, random forest had the best Brier score for the positive cases for all framing structures. These findings highlight the importance of clarifying the most important evaluation metric for each case and then testing different ML algorithms to find the one that optimizes the specific metric of interest. Take the case of dementia risk prediction, where a recent study evaluated the calibration of MLRP models to predict the risk of developing dementia the authors found that the models significantly overestimated the risk of developing dementia. At a predicted risk of 40%, the observed risk was only 10%—an overestimation of 30%. For models indented for individualized predictions an overestimation of this magnitude could lead to wrong decisions and well-calibrated estimates should have been a primary evaluation metric during model development^21,22^.

The frequency of predictions can have an impact on the clinical utility associated with the model because factors such as the number of false positive predictions could increase with frequency of use, influencing the clinical translation of the MLRP model.

An apparently good model with strong evaluation results on both discrimination and calibration is not necessarily usable. These profound consequences of framing of the MLRP model mandate attention from healthcare clinicians and data scientists: understanding and reporting framing are pivotal for the successful development and clinical implementation of future MLRP technology. Model framing must reflect the expected clinical environment. The importance of appropriate framing is by no means exclusive to sepsis prediction but applies to most clinical risk prediction models.

A major strength of the current study is that we created a platform for discussing the concept of framing as essential to building MLRP models among healthcare personal and data scientists. On a large, population-based, open cohort, we showed that the same ML architecture that was applied to the same data gave rise to many different models when the framing varied. We believe including clear framing details in future risk prediction models is fundamental to enabling healthcare professionals to discuss the clinical value of developed models or models under development with technical professionals who construct ML models. Thus, we encourage updating the “transparent reporting of a multivariable reduction model for individual prognosis or diagnosis” (TRIPOD) guidelines to include specific details about framing:

- For studies that use the framing structures, such as prediction windows and window shift, these must be reported to support a clear description of the source of data. Possibly as further elaboration under Item 4 in TRIPOD.
- A clear description of the logic behind “event-triggers” for actual clinical use with stated arguments—e.g., FP fatigue—either as a new item or elaboration to item 7a.

Especially (b) is important knowledge when assessing clinical utility. Both the sliding window variants and the on clinical demand structure can be seen as special cases of the at-event structure with different event-triggers. For the former with event = “every six hours”, and the latter event = “clinical demand”. An important point is that the event-trigger for a prediction in the framing formulation strongly affects the risk/inevitability of fatigue caused by false positives.

The current paper focuses on how MLRP models can be framed, assuming that the framing corresponds directly to how the model is evaluated. This need not be the case because the evaluation itself can be framed in different ways, just like framing the model. If a developer has trained a sequence-to-sequence MLRP model, he/she can choose to evaluate all timesteps as independent samples or only evaluate performance at specific timesteps relative to the event of interest. The former would yield an evaluation equivalent to if the framing structure of the model was the sliding window approach. The latter would yield an evaluation equivalent to the fixed time to onset structure.

Bedoya et al. fixed the number of alerts allowed per hour and reported the number of sepsis cases identified early per day to reflect the need to limit the number of alerts given to front-line clinicians^23^. To simulate a real-time scenario, evaluation metrics were calculated using the maximum score within windows ranging in size from 1 to 12 h. This kind of evaluation allowed the authors to plot the average cases detected early against the average alarms fired, which visualizes the threshold trade-off in a new way.

Somewhat related, Lauritsen et al. suggested a sequential evaluation that used the maximum probability of all predictions until the current timestep, such that model decisions in earlier timesteps were carried through and accounted for when evaluating model performance in subsequent timesteps^24^.

Similarly, Wong et al. suggested a hospitalization-level AUROC based on the entire trajectory of predictions to enable more realistic evaluations^25^.

Futoma et al. suggested a simulated real-time evaluation approach in which they defined a set of clinical rules for when a prediction should be considered true positive, false positive, and so on. One example was an alarm for a sepsis between 0 and 48 h before sepsis onset counting as a true positive. However, if the alarm went off more than 48 h in advance, it would be a false positive^16^.

Wiens et al. suggested a decision threshold based on the 90th percentile. In addition, the authors evaluated how far in advance their model could correctly identify positive cases. This analysis was visualized in a plot showing the fraction of identified cases against how many days in advance the prediction was made^19^.

Hyland et al. evaluated their proposed MLRP model, focusing on the percentage of events the system was able to detect against the rate of false alarms^26^. In doing so, they employed a silencing policy to reduce unnecessary repetitive alarms and investigated the effects of different silencing periods on the overall system performance.

The different framing structures led to a variation in class balance between 1:15 and 1:750 (Fig. 3). The derived clinical consequences of basing a model on unbalanced data could not be immediately observed using the AUROC, but the AUPRC varied greatly and provided a deeper understanding of the models’ performance (Fig. 3). Because precision recall curves do not use true negatives, the AUPRC will not be affected by a large proportion of true negatives in the data because it will focus on how the model handles a small fraction of positive examples. If the model handles the positive class well, the AUPRC will be high; if the model handles the positive class poorly, the AUPRC will be low^27^.

The on clinical demand framing, which involved samples for each time clinicians made an EWS assessment in the clinic, showed the lowest percentage of missing values among the vital sign parameters (Table 3); this model was also able to learn more temporal dependencies than the others.

In Fig. 5, the SHAP values are plotted against the SpO_2_ values in a SHAP dependence plot; the models have each learned to interpret SpO_2_ differently. The fixed time to onset model associates high SpO_2_ with the development of sepsis, which is, of course, wrong. This association might come from biases introduced in the framing and emphasizes the importance of both the right framing and need for transparent risk prediction models that can be verified by clinicians^6^.

The present study is not without limitations. It is a weakness that we did not test sequential models because they are widely applied in the sepsis prediction literature. However, even if we had included sequential models, they would still need to be framed and evaluated at specific times. In terms of the SHAP analysis, the addition of sequential models would lead to explanations over multiple timesteps that would have to be equated.

Another limitation is that the current study only concerned sepsis risk prediction in secondary care, and the findings may vary in different settings and for other outcomes. However, the fundamentals of how framing is directly linked to the clinical problem, which the ML model must solve, do not change.

When working with MLRP models developers first need to think carefully about how the MLRP model will be used in a clinical setting. What is the clinical question of interest, and how should the MLRP model be able to help in answering this question? Examples of such questions and the corresponding framing structure are given in Table 4.

**Table 4:** Examples of clinical questions and a corresponding framing.

Question (*Will this patient…)	When to predict	Framing structure	Lead window	Prediction window	Window Shift
*Develop sepsis within the next 12 h?	Each hour	SEQ/SW	NA	12 h	1 h
*Develop sepsis within the next 12 h?	Preoperatively	At event	NA	12 h	NA
*Experience a preventable hospitalization within 90 days?	Each week	SEQ/SW	NA	90 days	1 week
*Experience a preventable hospitalization within 90 days?	GP visits	At event	NA	90 days	NA
*Develop acute kidney injury during this hospitalization?	Admission	At event	NA	Entire admission	NA
*Need medical attention during the day tomorrow?	During round	At event/SW/SEQ	12 h	12 h	NA
How long does this patient have to live?	At diagnosis	Survival analysis	NA	Until study ends	NA
Expected P-sodium levels this time tomorrow	At home visit	OCD	18 h	12 h	NA

*SEQ* sequential, *SW* sliding window, *OCD* on clinical demand, *GP* general practitioner, *NA* not available.

A model to predict whether a patient will be admitted with a preventable hospitalization within some prediction window can easily be constructed so that predictions are made consecutively, such as every month. However, if the model is only used when the patient is in contact with the healthcare system, it makes much more sense to map the patient’s touch points with the healthcare system, such as GP visits or home care, and construct a dataset with predictions for each touch point. Such a change in the event-trigger for predictions will lead to fewer false positive predictions, while the model better represents the real use scenario.

When it comes to choosing the right lengths for different windows, there are no definitive rules, but there are some guidelines that can be useful. An observation window can have either a fixed or variable length. Variable length only makes sense if the developer intends to use a model architecture supporting masking, such as RNNs. If RNN techniques are not used, the observation window will typically be set to a specific size. Often, some manual feature engineering will be necessary, such as modeling the temporal development of parameters as differences between timesteps or trends. However, the size of the observation window may affect the signal-to-noise ratio of the representation of the explanatory variables. If the clinical signal being modeled is present only in the 6 h preceding an event, it makes little sense to have an observation window of 24 h unless valuable baseline information can be found in the first part of the observation window.

For many problems, the number of false positives ends up being a significant hindrance for further implementation, and often, it is not possible to improve the model’s performance to an acceptable level simply by applying more complicated models. Instead, it is recommended to think in terms of framing approaches to reduce the number of false positives, such as with a silencing policy. Basically, this comes down to (1) reducing the number of times the model is used or (2) changing the threshold for when the model produces a positive prediction.

Framing of the MLPR model at the first stage in development is crucial because all the subsequent work of training and evaluating will be in the context of how the model has been framed. Explicit documentation thereof will make it easier to compare the evaluation metrics among published studies. Our results illustrates that an apparently good model with strong evaluation results on both discrimination and calibration is not necessarily clinically usable. Hence, it is important to assess the objective evaluation results in the context of a more subjective evaluation of the way in which the model is framed. We recommend that problem framing be made very clear in future articles and suggest an update to the TRIPOD guidelines to include specific details about constructions in the framing structure, such as prediction windows and window shift as well as a description about event-triggers for a prediction that strongly affects the risk of clinician fatigue caused by false positives.

## Methods

### Data source

The dataset came from the CROSS-TRACKS cohort, a population-based, open cohort containing routinely collected data from primary and secondary healthcare partners, combined with data from national registries. A full description of the cohort has been published^28^, but in brief, the CROSS-TRACKS cohort consists of all citizens aged over 18 years and residing in the catchment area of Horsens Regional Hospital, a teaching hospital in the Central Denmark region, serving a population of 221,283 residents in four Danish municipalities (Odder, Hedensted, Skanderborg, and Horsens). The cohort entry date was September 1, 2012, and inclusion will last through 2022. Also, a 10-year look-back period and a 5-year follow-up period are included. The cohort offers a complete, multidimensional model of primary and secondary healthcare data, including data from municipalities. This multidimensionality is possible because of a merger of all the datasets via the unique personal identification number given to all Danish citizens^29^.

The study was approved by the Danish Data Protection Agency (case number 1-16-02-541-15). In addition, the data used were collected with the approval of the steering committee for CROSS-TRACKS. Only retrospective data were used, without the active involvement of patients or potential influence on their treatment. Therefore, under current national laws, no formal ethical approval was necessary.

### Study population

Outpatient contacts were not included. We excluded inpatient admissions shorter than 24 h or longer than 50 days. Excluding patients based on length of stay is not reproducible in a prospective setting, and it should be noted that we have used this exclusion requirement only as it is not intended to implement this model prospectively. In the study period, 19,976 inpatient admissions were applicable (for cohort characteristics, see Supplementary Table 2). The included admissions were distributed across 13,134 unique residents. The prevalence of sepsis among these admissions was 6.25%.

### Disease definition

We defined sepsis for individual cases using the recent Sepsis-3^2,30^, as also evaluated in a machine learning (ML) algorithm by Moor et al. ^9^, according to which both suspected infection and organ dysfunction needed to be present^2,9,30^. Suspected infection was defined by the coherent occurrence during the inpatient admission of (i) samples obtained (regardless of type) for culture purposes and (ii) antibiotic administration. When a sample was obtained for culture before the administration of antibiotics, the antibiotic had to be administered within 72 h. If the antibiotic was administered first, then the culture sample had to follow within 24 h. The degree of organ dysfunction was determined by an acute increase of two or more points in a SOFA score. In implementing the organ dysfunction criterion, we used a 72-h window from 48 h before to 24 h after the index time of suspected infection, as suggested earlier^9,30^. The Sepsis-3 implementation is illustrated in Supplementary Fig. 2.

### Framing of the prediction model

The temporal constructions, such as prediction times and window shifts, can be combined to form different framing structures, but it is only when these framing structures are implemented with specific choices regarding what to predict, when to predict and how often, that the actual framing of the MLRP model is formed. From the literature review above we identified eight different framing structures that apply to the sepsis risk prediction problem (Fig. 2). Four structures*, a–d*, were selected, and implemented, as examples that demonstrated the consequences of the framing of the MLRP model. The four chosen framing structures were selected by the authors because they were believed to represent realistic framings for a clinical deployment in our general ward setup:

- *Fixed time to onset* (Fig. 2a). The time of prediction for a sepsis-positive patient was fixed to 12 h before sepsis onset (lead window of 12 h, combined with a prediction window of only one point in time). For sepsis-negative patients, the “onset” time was set at a random time during admission. The fixed time to onset structure can be defined as an ‘at event’ structure with event = ‘12 h before onset’. We used an observation window of 12 h.
- *Sliding window* (Fig. 2b). The entire admission was split into chunks, for example, 8 h; each chunk was labeled sepsis positive if sepsis onset occurred within the prediction window and sepsis negative otherwise. We used a prediction window of 12 h, an observation window of 12 h and a window shift of 6 h.
- *Sliding window with dynamic inclusion* (Fig. 2c). The sampling started when a criterion was met, this case being a SOFA score of greater than 0. This structure differs from the *sliding window* approach by sampling fewer times and omitting samples known to be negative, thereby being a way to reduce the number of true negative samples in the dataset without reducing the true positives. We used a prediction window of 12 h, an observation window of 12 h and a window shift of 6 h.
- *On clinical demand* (Fig. 2d). Samples are only collected at points in time, where early warning score (EWS) assessments were performed by clinical staff. The sample interval is variable because the physician determines the rate of EWS assessments for a given patient on a daily basis. Samples are labeled sepsis positive if sepsis onset occurs within the prediction window and sepsis negative otherwise. The on clinical demand structure can be defined as an “at event” structure with event = “clinical demand”. We used a prediction window of 12 h, and an observation window of 12 h.

The remaining four structures, *e–h*, were not selected, because it was not a goal for the present study to test an exhaustive list of framing structures, but instead to demonstrate the importance of proper framing and documentation by showing the effect that a difference in MLRP model framings can have on conclusions in our general ward population. However, the four structures are still relevant for reference, as they could be optimal in other clinical settings and so, a description of these can be found in the Supplementary Note 1.

### Preprocessing

In the data extracted from the CROSS-TRACKS cohort, each sample is represented using 12-h observation windows with data for clinical parameters (19 blood tests and 6 vital signs, as shown in Table 5). The data from the 12-h observation window were extracted as two timesteps, each with a 6-h duration. In the case of multiple measurements within an hour, the mean was calculated and used in place of an individual measurement. A forward and backward imputation operation was performed to lower the number of missing values. Finally, the ∆ parameters (the difference between the two timesteps) were calculated to create a new set of parameters, doubling the total number of parameters to 50.

**Table 5:** List of clinical parameters.

Laboratory parameters		
P(aB)-Hydrogen carbonate	P(aB)-Potassium	P-Bilirubin
P(aB)-pO2	B-Leukocytes	P-Potassium
P(aB)-pCO2	B-Neutrophils	P-Glucose
P(aB)-pH	B-Platelets	P-C-reactive protein (CRP)
P(aB)-Lactate	P-Sodium	Glomerular filtration rate (eGFR)
P(aB)-Sodium	P-Albumin	
P(aB)-Chloride	P-Creatinine	

Vital sign parameters		
Systolic blood pressure	Respiratory frequency	SpO_2_ (Pulse oximetry)
Diastolic blood pressure	Pulse	Temperature

### Models

We used a set of five ML algorithms to build the MLRP models: Extremely randomized trees (Extra Trees Classifier)^31^, Random Forest Classifier^32^, Light Gradient Boosting Machine (LightGBM)^33^, Extreme Gradient Boosting (XGBoost)^34^, and Logistic Regression. For each of the four framing structures, we trained and tested the complete set of ML algorithms and repeated over five cross-validation folds, yielding a total of one hundred trained models. Details regarding implementation can be found in the linked GitHub repository.

### Evaluations

All risk prediction models were validated using fivefold cross-validation. Data were randomly divided into 5 portions of 20% each. For each fold, four portions (80%) were used to fit the risk prediction model parameters during training. The remaining 20% was split into 2 portions of 10% each for validation and testing. The validation data were used to perform an unbiased evaluation of model fit during training, and the test data were used to provide an unbiased evaluation of the final models. All data for each patient were assigned to training, validation, or test data. The cross-validation scheme is illustrated in Supplementary Fig. 3.

As comparative measures for discrimination, we used the area under the receiver operating characteristic curve (AUROC) and the area under the precision recall curve (AUPRC). The Brier score was used to assess discrimination and calibration in combination. We computed the stratified Brier score^35,36^ to enable a better assessment of how varying class imbalance affected the calibration. As demonstrated by Wallance et al., class probability estimates obtained in imbalanced scenarios systematically underestimate the probabilities for minority classes, despite having proper overall calibration^36^. However, because the Brier score is a combined measure, a lower Brier score does not necessarily imply better calibration^21,37^.

The average calibration error (ACE) was used as the primary metric for calibration. The ACE is calculated as follows: First, the predicted probabilities are discretized into a fixed number of bins. Second, the difference between the fraction of correct predictions and average of the probabilities in each bin is computed. Finally, the computed differences are combined through an unweighted average^38^. The equal weighting is favorable in imbalanced situations because the rightmost bins with more positive samples are weighted equal to the leftmost bins that contain most of the samples.

### Secondary analysis

For the secondary analysis, we used a combination of XGBoost^34^ and Shapley additive explanations SHAP^39^. XGBoost is a specific implementation of gradient tree boosting that produces a risk prediction model (called a strong learner) in the form of an ensemble of weak risk prediction models (weak learners), typically decision trees. The individual risk prediction models are built sequentially by adding several decision trees, each of which is trained to minimize the risk residuals for the previous model^39,40^. Further details about implementation can be found in the linked code repository.

SHAP is a game theoretic approach for explaining individual predictions in a risk prediction model. SHAP implements “Shapley values,” which define how to fairly distribute the payout among cooperative players in a game, where, in this setup, a prediction (value) corresponds to the payout and features correspond to players in the game. A more in-depth description for SHAP can be found in Supplementary Note 2.

### Reporting summary

Further information on research design is available in the Nature Research Reporting Summary linked to this article.

## Supplementary information

Supplementary Information

Reporting Summary

## Data Availability

The authors accessed the data referred to herein through the CROSS-TRACKS cohort, which is a newer Danish cohort combining primary and secondary sector data. Due to EU regulations, specifically the GDPR, these data are not readily available to the wider research community. However, all researchers can apply for access to the data by following the instructions on this page: http://www.tvaerspor.dk/.

## Code availability

The code used to conduct the analyses is shared here: https://github.com/SimonMeyerLauritsen/Framing, and can be reused without restrictions. For data extraction, our experimental framework for the data uses proprietary libraries that belong to Enversion A/S, and we are unable to publicly release this code. We have described the experiments and implementation details in the Methods section to allow for independent replication. Further inquiry regarding the specific nature of the ML models can be made by relevant parties to the corresponding author.
