# Supplementary material for: The Framing of machine learning risk prediction models illustrated by evaluation of sepsis in general wards
Source: NPJ Digit Med. 2021 Nov 15;4:158. doi: 10.1038/s41746-021-00529-x (PMC8593052; doi:10.1038/s41746-021-00529-x)
Supplement: Supplementary file 1 — Supplementary Information [file 41746_2021_529_MOESM1_ESM.pdf]

SUPPLEMENTARY INFORMATION FOR

The *Framing* of Machine Learning Risk Prediction Models Illustrated by Evaluation of Sepsis in General Wards

Simon Meyer Lauritsen,[1,2] Bo Thiesson,[1,3] Marianne Johansson Jørgensen,[4] Anders Hammerich Riis,[1] Ulrick Skipper Espelund,[4,5] Jesper Bo Weile,[6,7] Jeppe Lange[2,4]

[1]Enversion A/S, Fiskerivej 12, 1st floor, 8000 Aarhus C, Denmark; [2]Department of Clinical Medicine, Aarhus University, Aarhus N, Denmark; [3]Department of Engineering, Aarhus University, Aarhus C, Denmark; [4]Department of Research, Horsens Regional Hospital, Horsens, Denmark; Department of Anesthesiology, Horsens Regional Hospital, Denmark; [6]Emergency Department, Horsens Regional Hospital, Denmark; [7]Research Center for Emergency Medicine, Aarhus University Hospital, Denmark

**Corresponding author: Simon Meyer Lauritsen (sla@enversion.dk)*

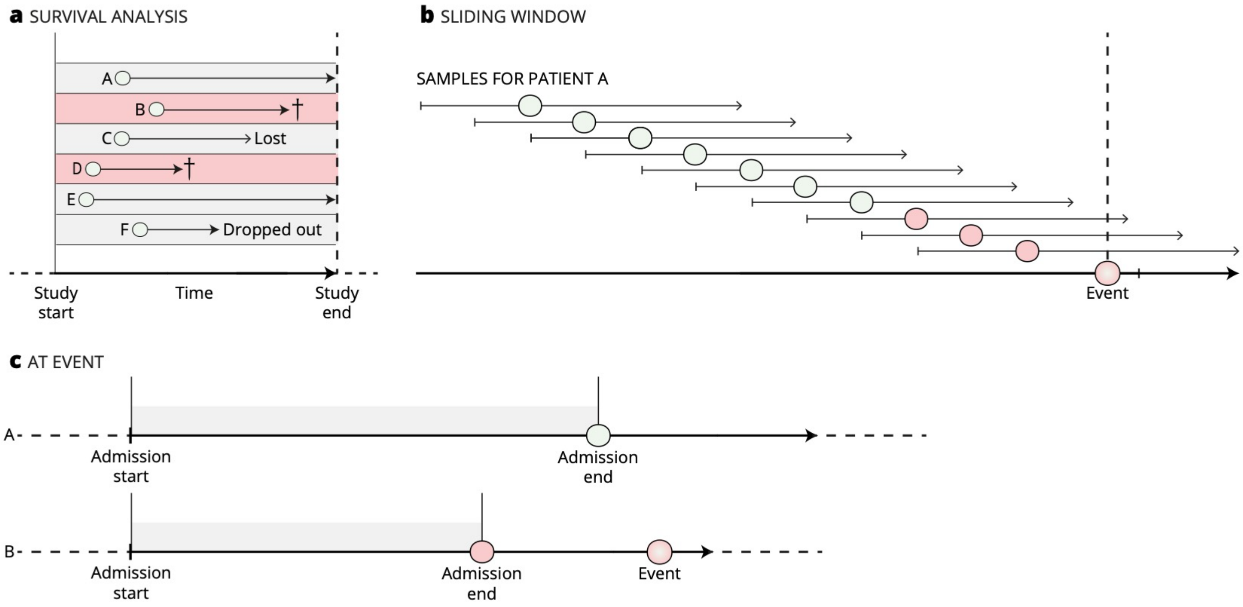

Supplementary Figure 1 | Three examples of popular framing approaches. (**a**) shows survival analysis, which is a time-to-event analysis that is used to investigate the length of time until the occurrence of some well-defined event of interest, such as mortality. A, B, C, D, E and F indicate different patients. (**b**) shows the sliding window approach, which is used to convert a sequential supervised learning problem into a standard supervised learning problem. (**c**) an at-event prediction, which is a left aligned way prediction at specific time points such as admission. A and B indicate different patients.

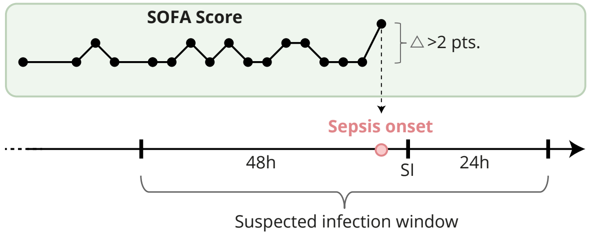

Supplementary Figure 2 | Sepsis definition. Figure showing how sepsis onset is defined. SOFA: Sequential Organ Failure

Assessment Scores, SI: Suspected Infection.

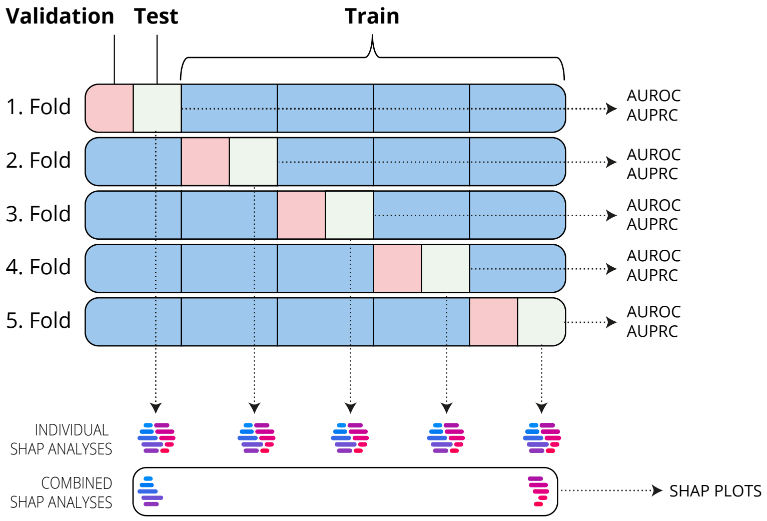

Supplementary Figure 3 | Cross-validation scheme. Data were randomly divided into 5 portions of 20% each. For each fold four portions (80 %) were used to fit the xAI-EWS model parameters during training. The remaining 20% was split into two portions of

10% each for validation and test. The validation data were used to perform an unbiased evaluation of a model fit during training, and the test data were used to provide an unbiased evaluation of the final model. For each fold data were shifted such that a new portion was used for testing. All data for a single patient was assigned to either train, validation or test data. SHAP analysis was conducted for each fold in cross validation, giving a total of five individual SHAP analyses. These five SHAP analyses were combined in one pooled analysis, which was the basis for the explanation results given in Figure 4 and Figure 5.

Supplementary Note 1: Description of framing approaches *e–h*.

- *Sequential approach with prediction window* (Figure 2e). Data for one patient were considered a single sample with multiple prediction times. This contrasts the sliding window approach, where each prediction time is considered an independent sample. The observation window expands as the patient is hospitalized for a longer period of time. The prediction window is of a fixed size and moves along with prediction time, generating a positive sample if sepsis occurs within the prediction window.

- *Sequential approach with entire admission as prediction window* (Figure 2f). This is similar to the sequential approach above, except the problem is not predicting whether sepsis onset will occur within the moving fixed size prediction window but if hospitalization will lead to sepsis *at any time*.

- *At event* (Figure 2g). Sample generation is linked to specific events, such as admission, preoperative assessment, or intubation.

- *Random time to onset* (Figure 2h). This is similar to the fixed time to onset approach except that the time of prediction for sepsis-positive samples is chosen randomly at some interval before sepsis onset.

Supplementary Note 2: Detailed explanation of how we used SHAP.
In SHAP, the risk prediction model is explained by declaring the parameters (i.e., covariate or confounder) or "players" in a game where the prediction is the "payout." SHAP values are at the same scale as the model outcome in the risk prediction—here, this would be the probability of sepsis. A negative SHAP value indicates a decreased probability of the outcome, and a positive value indicates increased probability. SHAP values sum to the difference between the predicted output for a given individual and the expected output of the model across the population. In other words, each SHAP value expresses the marginal effect that the observed parameter for the individual has on the final prediction rather than just predicting the prevalence.

One can explore each parameter's average impact on the prediction model by calculating the means of the absolute SHAP values across all individuals. Parameters with a high impact on the predicted outcome were identified by ranking these means by their magnitude (Figure 4). A plot was used to identify parameters of high importance for predicting the outcomes. The plot cannot show whether there are large effects of the outlying parameters on a few individuals because it only depicts the means of the absolute SHAP values.

A SHAP summary plot is a bird's eye view of parameter importance and what is driving it. The plot comprises many dots—one dot for each observed parameter for an individual in the investigated sample. The vertical location of the dot defines the parameter, and the horizontal position is the computed SHAP value for that parameter. The color shows the parameter value—low values are marked in blue, and high values are marked in red. Values in the middle are marked in purple. If a "swarm" of dots is centered around zero, the parameter has no effect on the model output.

For example, we can consider respiratory frequency, which is the most important parameter in Figure 3d. In general, the model associates high respiratory frequency (red) with an increase in the risk of sepsis and low

respiratory frequency (blue) with a low risk of sepsis. An average respiratory frequency (purple) is not associated with either a high or low risk of sepsis. The vertical extent of the swarm of dots correlates with the number of measurements, clearly showing that lower respiratory frequency measurements have been measured more than higher frequency ones.

The combination of XGBoost and SHAP is especially powerful because both can operate on data with missing values. One of the key characteristics of XGBoost is the sparsity awareness property, which allows XGBoost to process data with missing values without doing imputation first. During training, the optimal default direction is found by trying both directions in a split and choosing the one that leads to the highest gain. The SHAP summary displays missing values as gray dots, such that the SHAP effect of missing values can be analyzed.

Supplementary Table 1

Supplementary Table 1 – missing values for parameters used in the four models

Parameter	On clinical demand (%)	Sliding window (%)	Fixed time to onset (%)	Sliding window w. dynamic incl. (%)
Heart rate	0,00	36,61	24,97	34,47
Respiratory Frequency	0,00	38,85	26,65	36,81
SpO2	0,00	36,70	25,06	34,27
Systolic BP	0,00	36,45	24,77	34,29
Diastolic BP	0,07	36,51	24,88	34,36
Temperature	5,88	42,62	31,59	40,52
Heart rate Δ	26,81	80,86	71,48	78,30
Respiratory Frequency Δ	26,81	81,42	72,03	78,96
SpO2 Δ	26,81	80,91	71,53	78,30
Systolic BP Δ	26,81	80,79	71,33	78,21
Diastolic BP Δ	26,85	80,80	71,37	78,22
Temperature Δ	30,95	82,42	73,71	79,99
P-Sodium	71,77	69,95	60,99	66,54
P-Potassium	71,84	70,01	61,14	66,63
P-Creatinine	71,96	70,12	61,39	66,72
P-Albumin	73,47	70,95	62,27	67,65
B-Leukocytes	73,59	72,27	62,14	69,49
P-C-reactive protein	73,92	72,78	63,71	69,92
P-Sodium Δ	75,90	89,21	84,55	87,01
P-Potassium Δ	75,93	89,23	84,62	87,03
P-Creatinine Δ	76,05	89,26	84,74	87,07
P-Albumin Δ	77,27	89,63	85,07	87,51
P-C-reactive protein Δ	77,71	90,36	85,87	88,42
B-Leukocytes Δ	78,20	90,29	84,83	88,37
eGFR	81,39	79,56	74,72	74,70
P-Glucose	82,17	79,15	83,14	75,21
P-Glucose Δ	83,33	88,69	89,45	86,19
eGFR Δ	84,11	92,33	89,45	89,99
B-Platelets	90,28	95,00	93,93	94,09
P-Bilirubine	90,85	94,88	93,20	93,97
P(aB)-pH	92,15	93,91	94,65	91,67
P(aB)-pCO2	92,15	93,91	94,64	91,67
P(aB)-pO2	92,16	93,93	94,65	91,70
P(aB)-Lactate	92,18	93,94	94,67	91,72
P(aB)-Hydrogen carbonate	92,22	93,93	94,66	91,71
P(aB)-Potassium	92,35	94,07	94,75	91,90
P(aB)-Sodium	92,36	94,08	94,76	91,91
B-Platelets Δ	92,86	97,39	96,79	96,73
P-Bilirubine Δ	92,88	97,71	96,84	97,14
P(aB)-pCO2 Δ	93,59	95,51	95,79	93,83
P(aB)-pH Δ	93,59	95,50	95,80	93,83
P(aB)-pO2 Δ	93,61	95,52	95,80	93,84
P(aB)-Lactate Δ	93,62	95,52	95,81	93,85
P(aB)-Hydrogen carbonate Δ	93,65	95,52	95,80	93,85
P(aB)-Potassium Δ	93,78	95,60	95,87	93,96
P(aB)-Sodium Δ	93,79	95,60	95,88	93,97

P(aB)-Chloride	97,98	98,64	98,55	98,20
P(aB)-Chloride Δ	98,46	98,96	98,84	98,62
B-Neutrophils	99,98	99,99	99,96	99,98
B-Neutrophils Δ	99,98	99,99	99,97	99,99

Supplementary Table 2

Supplementary table 2 – cohort characteristic

Characteristic	Sepsis negative	Sepsis positive	Total
**Number of patients**	18,726 (94)	1,250 (6)	19,976 (100)
**Age**			
Age 0-17	77 (0)	0 (0)	77 (0)
Age 18-39	1,460 (8)	55 (4)	1,515 (8)
Age 40-64	4,347 (23)	283 (23)	4,630 (23)
Age 65-79	6,921 (37)	522 (42)	7,443 (37)
Age 80+	5,921 (32)	390 (31)	6,311 (32)
**Age Non-missing N (%)**	18,726 (100)	1,250 (100)	19,976 (100)
**Age Mean (SD)**	70 (17)	71 (15)	70 (17)
**Age Median (IQR)**	73 (61-83)	73 (64-82)	73 (61-82)
**Gender**			
Female	9,844 (53)	488 (39)	10,332 (52)
Male	8,882 (47)	762 (61)	9,644 (48)
**Municipality**			
Hedensted	3,956 (21)	277 (22)	4,233 (21)
Horsens	9,098 (49)	590 (47)	9,688 (48)
Odder	2,014 (11)	133 (11)	2,147 (11)
Other	37 (0)	1 (0)	38 (0)
Skanderborg	3,621 (19)	249 (20)	3,870 (19)
**Marital status**			
Divorced or dissolved partnership	2,644 (14)	187 (15)	2,831 (14)
Married or registered partnership	8,070 (43)	577 (46)	8,647 (43)
Not married	1,721 (9)	89 (7)	1,810 (9)
Unknown	1,122 (6)	87 (7)	1,209 (6)
Widowed or longest living in a registered partnership	5,169 (28)	310 (25)	5,479 (27)
**Cohabitation status: Living alone**	7,397 (40)	499 (40)	7,896 (40)
**Socioeconomic status (within 1 year prior to hospitalisation)**			
Health-related benefit	2,799 (15)	186 (15)	2,985 (15)
Labour-market-related benefit	514 (3)	31 (2)	545 (3)
Normal retirement	4,323 (23)	321 (26)	4,644 (23)
Self-supporting	11,090 (59)	712 (57)	11,802 (59)
**Health services from the municipalities (within 30 days prior to hospitalisation)**			
Rehabilitation (eg physiotherapy)			
No	15,394 (82)	1,038 (83)	16,432 (82)
Yes	3,332 (18)	212 (17)	3,544 (18)
Practical help (eg home cleaning)			
No	11,984 (64)	826 (66)	12,810 (64)
Yes	6,742 (36)	424 (34)	7,166 (36)
Personal care (eg weekly bath)			
No	11,413 (61)	784 (63)	12,197 (61)
Yes	7,313 (39)	466 (37)	7,779 (39)
Home visits by a community nurse (eg injection of medicine)			
No	10,433 (56)	697 (56)	11,130 (56)
Yes	8,293 (44)	553 (44)	8,846 (44)
Any service			
No	8,525 (46)	566 (45)	9,091 (46)
Yes	10,201 (54)	684 (55)	10,885 (54)
**Health services from the municipalities (within 30 days prior to hospitalisation)**			
1 Home visits by a community nurse	8,293 (44)	553 (44)	8,846 (44)
2 Personal care	1,008 (5)	68 (5)	1,076 (5)
3 Practical help	615 (3)	40 (3)	655 (3)
4 Rehabilitation	285 (2)	23 (2)	308 (2)
5 No service	8,525 (46)	566 (45)	9,091 (46)
**Health services from the municipalities (within 90 days prior to hospitalisation)**			
1 Home visits by a community nurse	8,625 (46)	582 (47)	9,207 (46)
2 Personal care	1,004 (5)	64 (5)	1,068 (5)
3 Practical help	625 (3)	42 (3)	667 (3)

4 Rehabilitation	356 (2)	29 (2)	385 (2)
5 No service	8,116 (43)	533 (43)	8,649 (43)
**Primary healthcare services (within 30 days prior to hospitalisation)**			
Daytime face-to-face contact			
No	5,286 (28)	358 (29)	5,644 (28)
Yes	13,440 (72)	892 (71)	14,332 (72)
Out-of-hours face-to-face contact			
No	16,500 (88)	1,115 (89)	17,615 (88)
Yes	2,226 (12)	135 (11)	2,361 (12)
Any GP contact			
No	1,283 (7)	88 (7)	1,371 (7)
Yes	17,443 (93)	1,162 (93)	18,605 (93)
Dentist (within 1 year)			
No	9,240 (49)	646 (52)	9,886 (49)
Yes	9,486 (51)	604 (48)	10,090 (51)
**Known in the primary setting (within 30 days prior to hospitalisation)**			
No	1,066 (6)	69 (6)	1,135 (6)
Yes	17,660 (94)	1,181 (94)	18,841 (94)
**Primary healthcare services (within 90 days prior to hospitalisation)**			
Daytime face-to-face contact			
No	2,448 (13)	166 (13)	2,614 (13)
Yes	16,278 (87)	1,084 (87)	17,362 (87)
Out-of-hours face-to-face contact			
No	15,850 (85)	1,073 (86)	16,923 (85)
Yes	2,876 (15)	177 (14)	3,053 (15)
Any GP contact			
No	464 (2)	29 (2)	493 (2)
Yes	18,262 (98)	1,221 (98)	19,483 (98)
**Known in the primary setting (within 90 days prior to hospitalisation)**			
No	394 (2)	25 (2)	419 (2)
Yes	18,332 (98)	1,225 (98)	19,557 (98)
**Charlson comorbidity index score (within 10 years prior to hospitalisation)**			
Score 0: No comorbidity	8,142 (43)	467 (37)	8,609 (43)
Score 1: Low comorbidity	4,032 (22)	261 (21)	4,293 (21)
Score 2: Medium comorbidity	2,667 (14)	218 (17)	2,885 (14)
Score 3+ High comorbidity	3,885 (21)	304 (24)	4,189 (21)
**Psychiatric disease (within 10 years prior to hospitalisation)**			
Mild psychiatric disease	4,042 (22)	262 (21)	4,304 (22)
No disease	14,219 (76)	957 (77)	15,176 (76)
Severe psychiatric disease	465 (2)	31 (2)	496 (2)
**BMI measurement**			
1 Within 30 days prior to the index date	15,742 (84)	1,006 (80)	16,748 (84)
2 Within 30 days after the index date	1,353 (7)	186 (15)	1,539 (8)
3 Within 180 to 30 days prior to the index date	511 (3)	13 (1)	524 (3)
4 Outside prioritized periods	636 (3)	24 (2)	660 (3)
5 No test	484 (3)	21 (2)	505 (3)
**BMI**			
BMI 0 to <18.5 kg/m2	1,432 (8)	72 (6)	1,504 (8)
BMI 18.5 to <25 kg/m2	7,323 (39)	475 (38)	7,798 (39)
BMI 25 to <30 kg/m2	5,183 (28)	384 (31)	5,567 (28)
BMI 30 to <35 kg/m2	2,327 (12)	185 (15)	2,512 (13)
BMI 35 to <40 kg/m2	850 (5)	63 (5)	913 (5)
BMI >= 40 kg/m2	491 (3)	26 (2)	517 (3)
Not measured	1,120 (6)	45 (4)	1,165 (6)
**BMI Non-missing N (%)**	17,606 (94)	1,205 (96)	18,811 (94)
**BMI Mean (SD)**	26 (6)	26 (6)	26 (6)
**BMI Median (IQR)**	25 (22-29)	26 (22-29)	25 (22-29)
**Alcohol intake registration**			
1 Within 30 days prior to the index date	4,699 (25)	291 (23)	4,990 (25)
2 Within 30 days after the index date	1,358 (7)	101 (8)	1,459 (7)
3 Within 180 to 30 days prior to the index date	1,826 (10)	110 (9)	1,936 (10)
4 Outside prioritized periods	5,268 (28)	359 (29)	5,627 (28)
5 No test	5,575 (30)	389 (31)	5,964 (30)
**Alcohol**			
Alcohol intake above recommandations	2,039 (11)	119 (10)	2,158 (11)
Alcohol intake within recommandations	5,844 (31)	383 (31)	6,227 (31)
No reported alcohol intake	10,843 (58)	748 (60)	11,591 (58)
**Smoking status registration**			

1 Within 30 days prior to the inclusion date	3,536 (19)	249 (20)	3,785 (19)
2 Within 30 days after the inclusion date	1,262 (7)	70 (6)	1,332 (7)
3 Within 180 to 30 days prior to the inclusion date	2,630 (14)	148 (12)	2,778 (14)
4 Outside prioritized periods	6,323 (34)	434 (35)	6,757 (34)
5 No test	4,975 (27)	349 (28)	5,324 (27)
**Smoking status**			
Current smoker	1,915 (10)	125 (10)	2,040 (10)
No smoking status reported	11,298 (60)	783 (63)	12,081 (60)
Non-smoker	2,195 (12)	124 (10)	2,319 (12)
Occational smoker	91 (0)	7 (1)	98 (0)
Previous smoker	3,227 (17)	211 (17)	3,438 (17)
**Smoking status**			
No	13,493 (72)	907 (73)	14,400 (72)
Yes	5,233 (28)	343 (27)	5,576 (28)
**Smoking status any time before hospitalisation**			
Current smoker	3,367 (18)	221 (18)	3,588 (18)
No smoking status reported	8,022 (43)	543 (43)	8,565 (43)
Non-smoker	2,818 (15)	161 (13)	2,979 (15)
Occational smoker	152 (1)	13 (1)	165 (1)
Previous smoker	4,367 (23)	312 (25)	4,679 (23)
**Smoking status any time before hospitalisation**			
No	10,840 (58)	704 (56)	11,544 (58)
Yes	7,886 (42)	546 (44)	8,432 (42)
**Diastolic blood pressure registration**			
1 Within 30 days prior to the inclusion date	18,558 (99)	1,220 (98)	19,778 (99)
2 Within 30 days after the inclusion date	51 (0)	28 (2)	79 (0)
3 Within 180 to 30 days prior to the inclusion date	41 (0)	0 (0)	41 (0)
4 Outside prioritized periods	47 (0)	2 (0)	49 (0)
5 No test	29 (0)	0 (0)	29 (0)
**Diastolic blood pressure (mmHg) Non-missing N (%)**	18,650 (100)	1,248 (100)	19,898 (100)
**Diastolic blood pressure (mmHg) Mean (SD)**	75 (13)	72 (14)	75 (13)
**Diastolic blood pressure (mmHg) Median (IQR)**	74 (66-83)	70 (61-80)	74 (65-83)
**Systolic blood pressure registration**			
1 Within 30 days prior to the inclusion date	18,558 (99)	1,220 (98)	19,778 (99)
2 Within 30 days after the inclusion date	51 (0)	28 (2)	79 (0)
3 Within 180 to 30 days prior to the inclusion date	41 (0)	0 (0)	41 (0)
4 Outside prioritized periods	47 (0)	2 (0)	49 (0)
5 No test	29 (0)	0 (0)	29 (0)
**Systolic blood pressure (mmHg) Non-missing N (%)**	18,650 (100)	1,248 (100)	19,898 (100)
**Systolic blood pressure (mmHg) Mean (SD)**	133 (22)	127 (25)	133 (23)
**Systolic blood pressure (mmHg) Median (IQR)**	131 (117-147)	124 (110-142)	130 (116-147)
**High blood pressure (diastolic > 90 mm Hg or systolic > 140 mm Hg)**			
No	11,969 (64)	889 (71)	12,858 (64)
Yes	6,757 (36)	361 (29)	7,118 (36)
**Use of prescription medicine (within 180 days prior to hospitalisation)**			
Antidiabetics (A10A, A10B)	3,236 (17)	252 (20)	3,488 (17)
Blood pressure medication (C02, C07, C08, C09)	10,281 (55)	765 (61)	11,046 (55)
Antibiotics (J01)	9,396 (50)	607 (49)	10,003 (50)
Inhaled corticosteroid therapy (R03)	4,524 (24)	297 (24)	4,821 (24)
Lipid-lowering treatment (C10)	6,170 (33)	483 (39)	6,653 (33)
Low dose aspirin (B01AC06 75, 100, 150 mg, N02BA01 100 mg)	4,887 (26)	377 (30)	5,264 (26)
**Hospital contacts (within 30 days prior to hospitalisation)**			
At least one hospital contact	17,594 (94)	1,067 (85)	18,661 (93)
At least one inpatient hospital contact	15,747 (84)	937 (75)	16,684 (84)
At least one inpatient hospital contact with acute admission	15,431 (82)	903 (72)	16,334 (82)
At least one outpatient hospital contact	6,420 (34)	416 (33)	6,836 (34)
At least one emergency room visit	3,613 (19)	202 (16)	3,815 (19)

Data are given as number (percentage) of patients, unless otherwise specified.
