# Supplementary material for: The Framing of machine learning risk prediction models illustrated by evaluation of sepsis in general wards
Source: NPJ Digit Med. 2021 Nov 15;4:158. doi: 10.1038/s41746-021-00529-x (PMC8593052; doi:10.1038/s41746-021-00529-x)
Supplement: Supplementary file 2 — Reporting Summary [file 41746_2021_529_MOESM2_ESM.pdf]

nature research

Corresponding author(s): Simon Meyer Lauritsen

Last updated by author(s): Oct 6, 2021

Reporting Summary

Nature Research wishes to improve the reproducibility of the work that we publish. This form provides structure for consistency and transparency in reporting. For further information on Nature Research policies, see our Editorial Policies and the Editorial Policy Checklist.

Statistics

For all statistical analyses, confirm that the following items are present in the figure legend, table legend, main text, or Methods section.

n/a	Confirmed	
☐	☒	The exact sample size (*n*) for each experimental group/condition, given as a discrete number and unit of measurement
☐	☒	A statement on whether measurements were taken from distinct samples or whether the same sample was measured repeatedly
☒	☐	The statistical test(s) used AND whether they are one- or two-sided<br>*Only common tests should be described solely by name; describe more complex techniques in the Methods section.*
☐	☒	A description of all covariates tested
☒	☐	A description of any assumptions or corrections, such as tests of normality and adjustment for multiple comparisons
☐	☒	A full description of the statistical parameters including central tendency (e.g. means) or other basic estimates (e.g. regression coefficient) AND variation (e.g. standard deviation) or associated estimates of uncertainty (e.g. confidence intervals)
☒	☐	For null hypothesis testing, the test statistic (e.g. *F*, *t*, *r*) with confidence intervals, effect sizes, degrees of freedom and *P* value noted<br>*Give P values as exact values whenever suitable.*
☒	☐	For Bayesian analysis, information on the choice of priors and Markov chain Monte Carlo settings
☒	☐	For hierarchical and complex designs, identification of the appropriate level for tests and full reporting of outcomes
☒	☐	Estimates of effect sizes (e.g. Cohen's *d*, Pearson's *r*), indicating how they were calculated

Our web collection on statistics for biologists contains articles on many of the points above.

Software and code

Policy information about availability of computer code

Data collection	The authors have accessed the data referred to herein by applying the CROSS-TRACKS cohort (see cohort profile: https://www.medrxiv.org/content/10.1101/2020.05.13.20100263v1), which is a newer Danish cohort that combines primary and secondary sector data. Due to the EU regulations, national law, and GDPR, these data are not readily available to the wider research community per se. However, all researchers can apply for access to the data by following the instructions on this page: http://www.tvaerspor.dk/.
Data analysis	We made use of several open-source libraries to conduct our experiments: The models used the the machine learning framework TensorFlow library with custom extensions (https://www.tensorflow.org) and Keras (https://keras.io). Explanations were calculated with the high-level library for explaining neural networks iNNvestigate (https://github.com/albermax/innvestigate). SHAP (https://github.com/slundberg/shap) with custom extensions was used to visualize explanations. Analysis was performed with custom code written in Python 3.5. Our experimental framework makes use of proprietary libraries that belong to Enversion A/S, and we are unable to publicly release this code. We have described the experiments and implementation details in the Methods section to allow for independent replication. Further inquiry regarding the specific nature of the AI model can be made by relevant parties to the corresponding author.

For manuscripts utilizing custom algorithms or software that are central to the research but not yet described in published literature, software must be made available to editors and reviewers. We strongly encourage code deposition in a community repository (e.g. GitHub). See the Nature Research guidelines for submitting code & software for further information.

Data

Policy information about availability of data

All manuscripts must include a data availability statement. This statement should provide the following information, where applicable:

- Accession codes, unique identifiers, or web links for publicly available datasets
- A list of figures that have associated raw data
- A description of any restrictions on data availability

The authors have accessed the data by applying the CROSS-TRACKS cohort, which is a newer Danish cohort that combines primary and secondary sector data. As with most register data/EHR data/health data we, as investigators, do not own data, and is not able to share data. This is both due to the EU regulations, national law and GDPR. However, all researchers can apply for access to the data by following the instructions on this page: http://www.tvaerspor.dk/.

Field-specific reporting

Please select the one below that is the best fit for your research. If you are not sure, read the appropriate sections before making your selection.

☒ Life sciences ☐ Behavioural & social sciences ☐ Ecological, evolutionary & environmental sciences

For a reference copy of the document with all sections, see nature.com/documents/nr-reporting-summary-flat.pdf

Life sciences study design

All studies must disclose on these points even when the disclosure is negative.

Sample size	In this study, we analyzed the secondary healthcare data of all residents of four Danish municipalities (Odder, Hedensted, Skanderborg, and Horsens) who were 18 years of age or older for the period of 2012–2017. The data contained information from the electronic health record (EHR), including biochemistry, medicine, microbiology, and procedure codes, and was extracted from the "CROSS-TRACKS" cohort, which embraces a mixed rural and urban multi-center population with four regional hospitals and one larger university hospital. Each hospital comprises multiple departmental units, such as emergency medicine, intensive care, and thoracic surgery. We included all 163,050 available inpatient admissions (45.86% male) during the study period and excluded only outpatient admissions. The included admissions were distributed across 66,288 unique residents. The prevalence for sepsis, AKI, and ALI among these admissions was 2.44%, 0.75%, and 1.68%, respectively (see Table 2).
Data exclusions	No data excluded.
Replication	In order to quantify reproducibility all analysis was data were randomly divided into 5 portions of 20% each. For each fold four portions (80 %) was used to fit the xAI-EWS model parameters during training. The remaining 20% was split into two portions of 10% each for validation and test. This allowed to report means values along with confidence intervals to indicate variation between experiments/folds. All patients in the test set were randomly selected and were not correlated in any way. Results were consistent between folds and can be observed in Figure 2. The cross validation scheme is illustrated in Supplementary Figure 1. Also, in the Supplementary information Table 1 and 2 all the raw output the evaluation is listed.
Randomization	Data were randomly divided into 5 portions of 20% each. For each fold four portions (80 %) was used to fit the xAI-EWS model parameters during training. The remaining 20% was split into two portions of 10% each for validation and test. The validation data were used to perform an unbiased evaluation of a model fit during training, and the test data were used to provide an unbiased evaluation of the final model. For each fold data were shifted such that a new portion was used for testing. All data for a single patient was assigned to either train, validation or test data.
Blinding	All data for a single patient was assigned to either train, test or validation splits randomly. After random assignment patient identification keys were removed, leaving only a cleaned dataset with only parameters and labels visible to the investigators. The entire proces of random assignment, training and testing was done in an automatic pipeline with no human interaction until test results were output.

Reporting for specific materials, systems and methods

We require information from authors about some types of materials, experimental systems and methods used in many studies. Here, indicate whether each material, system or method listed is relevant to your study. If you are not sure if a list item applies to your research, read the appropriate section before selecting a response.

Materials & experimental systems

n/a	Involved in the study	
☒	☐	Antibodies
☒	☐	Eukaryotic cell lines
☒	☐	Palaeontology and archaeology
☒	☐	Animals and other organisms
☐	☒	Human research participants
☒	☐	Clinical data
☒	☐	Dual use research of concern

Methods

n/a	Involved in the study	
☒	☐	ChIP-seq
☒	☐	Flow cytometry
☒	☐	MRI-based neuroimaging

Human research participants

Policy information about studies involving human research participants

Population characteristics	In this study, we analyzed the secondary healthcare data of all residents of four Danish municipalities (Odder, Hedensted, Skanderborg, and Horsens) who were 18 years of age or older for the period of 2012–2017. We included all 163,050 available inpatient admissions (45.86% male) during the study period and excluded only outpatient admissions.
Recruitment	The authors have accessed the data referred to herein by applying the CROSS-TRACKS cohort, which is a newer Danish cohort that combines primary and secondary sector data.
Ethics oversight	The study was approved by The Danish Data Protection Agency [case number 1-16-02-541-15]. Additionally, the data used in this work were collected with the approval of the steering committee for CROSS-TRACKS. Only retrospective data were used for this research without the active involvement of patients or potential influence on their treatment. Therefore, under the current national legislature, no formal ethical approval was necessary.

Note that full information on the approval of the study protocol must also be provided in the manuscript.
